# Independent evaluation of a canine Echinococcosis Control Programme in Hobukesar County, Xinjiang, China

**DOI:** 10.1016/j.actatropica.2015.01.009

**Published:** 2015-05

**Authors:** Freya van Kesteren, Xinwei Qi, Jiang Tao, Xiaohui Feng, Alexander Mastin, Philip S. Craig, Dominique A. Vuitton, Xinyu Duan, Xiangdong Chu, Jinlong Zhu, Hao Wen

**Affiliations:** a Cestode Zoonoses Research Group, School of Environment and Life Sciences, University of Salford, M5 4WT Salford, UK; b State Key Lab Incubation Base for Xinjiang Major Diseases Research and Xinjiang Key Laboratory of Echinococcosis, the First Affiliated Hospital of Xinjiang Medical University, Urumqi, Xinjiang, China; c WHO-Collaborating Centre for Prevention and Treatment of Human Echinococcosis, France; d People's Hospital of Hobukesar County, Hobukesar, Xinjiang, China

**Keywords:** *Echinococcus granulosus*, Control programme, Domestic dogs, Lot Quality Assurance Sampling, Xinjiang

## Abstract

The Xinjiang Uyghur Autonomous Region in northwest China is one of the world's most important foci for cystic echinococcosis. Domestic dogs are the main source for human infection, and previous studies in Xinjiang have found a canine *Echinococcus* spp. coproELISA prevalence of between 36% and 41%. In 2010 the Chinese National Echinococcosis Control Programme was implemented in Xinjiang, and includes regular dosing of domestic dogs with praziquantel. Six communities in Hobukesar County, northwest Xinjiang were assessed in relation to the impact of this control programme through dog necropsies, dog *Echinococcus* spp. coproantigen surveys based on Lot Quality Assurance Sampling (LQAS) and dog owner questionnaires. We found that 42.1% of necropsied dogs were infected with *Echinococcus granulosus*, and coproELISA prevalences were between 15% and 70% in the communities. Although approximately half of all dog owners reported dosing their dogs within the 12 months prior to sampling, coproELISA prevalence remained high. Regular praziquantel dosing of owned dogs in remote and semi-nomadic communities such as those in Hobukesar County is logistically very difficult and additional measures should be considered to reduce canine echinococcosis.

## Highlights

• Xinjiang is one of the most important foci of human cystic echinococcosis in the world.
• A control programme, including PZQ dosing of dogs, began in Xinjiang in 2010.
• The control programme was evaluated using LQAS sampling, dog necropsies and questionnaires.
• The control programme did not meet our evaluation criteria in most communities studied.
• Other measures should be considered to improve *Echinococcus* spp. control in Xinjiang.

## Graphical abstract

**Figure:**
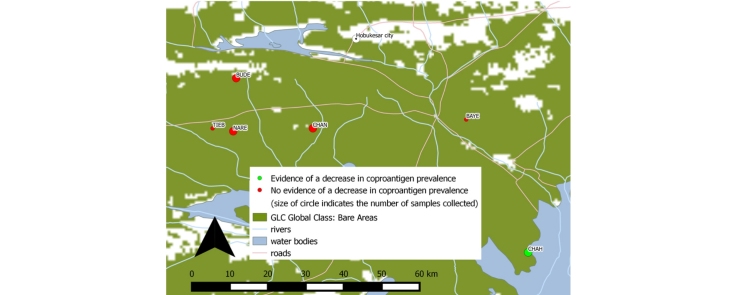

## 1 Introduction

The Xinjiang Uyghur Autonomous Region (Xinjiang) is an autonomous region of the People's Republic of China, located in the northwest of the country (Bart et al., 2006). Xinjiang is a multi-ethnic province, with ethnic groups including Uyghur, Han Kazakh, Hui and Mongol (Wang et al., 2001). Many people in north-western Xinjiang live in pastoral areas and have traditional (semi) nomadic lifestyles (Wang et al., 2001).

Xinjiang is one of the most important foci of human cystic echinococcosis in China and the world (Bart et al., 2006), and surveys in Hobukesar Mongol Autonomous County (Hobukesar County) in north-west Xinjiang found a human CE prevalence by ultrasound of 2.7% (Wang et al., 2005). Previous dog surveys in Hobukesar County have found necropsy and coproELISA prevalences of 36% (Wang et al., 2005), and a study conducted in Fuhai and Emin counties in north Xinjiang found that 41.2% of dogs were coproELISA positive for *Echinococcus* spp. (Wei et al., 2005). In 2006, the Chinese government implemented the National Echinococcosis Control Programme in Sichuan Province, and in 2010 this programme was expanded to include other provinces in China, including Xinjiang (WHO, 2011).

The Echinococcosis Control Programme aimed to achieve monthly praziquantel dosing of domestic dogs (Chinese Ministry of Health, 2007), as well as identifying human cases through ultrasound screening and subsequent medical treatment of patients (WHO, 2010). Specific methods proposed for reducing canine echinococcosis included registering all owned dogs in endemic areas, and deworming dogs using praziquantel (0.2 g/tablet), with 1–2 tablets administered to dogs weighing more than 15 kg. A de-worming frequency of once a month was aimed for, involving supervised dosing with praziquantel in baits. Workers dosing dogs should confirm the tablets were swallowed and the date of de-worming recorded on the dog registration card. The Control Programme also aimed to collect dog faeces 5 days after de-worming and bury or burn these to prevent environmental contamination, as well as taking measures to control dog numbers such as culling stray dogs (Chinese Ministry of Health, 2007). In April 2013 we visited six rural communities in Hobukesar County in north-west Xinjiang to assess the impact of the Control Programme in this County.

## 2 Materials and methods

### 2.1 Communities

Six communities in Hobukesar County were included: Narenhebuke (46.47°, 85.30°), Budengjian (46.65°, 85.31°), Changan Kul (46.48°, 85.57°), Chahete (46.06°, 86.30°), Bayenoma (46.51°, 86.09°) and Tiebukenwusan (46.48°, 85.23°). These communities included ethnic Mongolians, Kazakhs, and Han Chinese, and were based around livestock husbandry, although Chahete was established in 2010 as an agricultural community and consisted mostly of ethnic Han people that were relocated from Gansu and Sichuan provinces.

### 2.2 Dog necropsies

Thirty-eight unwanted or stray dogs were provided by a local dog catcher, including from Bayenoma (*n* = 3), Narenhebuke (*n* = 4), Changan Kul (*n* = 16), and three other County villages called Yikewutubulage (*n* = 9), Mogete (*n* = 2) and Busitinge (*n* = 1), with the locations of three dogs not recorded. All dogs were adults (estimated to be at least 1 year old). Twenty-one were male, and 17 were female. Dogs were captured alive and euthanised by a qualified animal technician (JT) using intravenous ketamine. The small intestine of each dog was removed post-mortem and inspected in the field by experienced researchers (PSC&JT) using a magnifying glass. Dogs were scored as *Echinococcus* spp. and *Taenia* spp. present/absent, with worm burdens estimated for *Echinococcus* spp. and counted for *Taenia* spp. Tapeworms were washed in water and stored in 70% ethanol for DNA analysis. Faecal samples were collected per rectum post-mortem and stored in 0.3% PBS Tween with 10% formalin for coproELISA testing. All samples were transported to Salford University, UK, at room temperature.

### 2.3 Lot Quality Assurance Sampling (LQAS)

LQAS is a form of stratified sampling which requires a relatively low number of samples whilst retaining a statistical basis (the small sample size required sometimes leads to misunderstanding of the statistical basis of LQAS, as described by Pagano and Valadez (2010). Although originally developed for quality evaluation in the manufacturing industry (Dodge and Romig, 1929), LQAS has more recently been applied to studies on disease and healthcare (for a review see Robertson and Valadez, 2006). The central concept of LQAS is the classification of ‘supervision areas’ (e.g. villages) in a dichotomous fashion – according to whether a target has been achieved – rather than attempting to present prevalence estimates for each area. For the purposes of the current study, a simplified form of LQAS was used, which requires only one input; the minimum ‘threshold’ prevalence of the outcome of interest which could be considered a ‘success’ or ‘failure’. The binomial distribution can then be used to estimate the cumulative probability distribution of the expected number of positive outcomes for a small sample size (often set at 19), given that the prevalence is at this stated threshold. From this, the minimum number of expected positive outcomes which gives a cumulative probability of greater than 0.1 can be estimated – known as the ‘decision rule’. If the number of positive individuals in a sample is lower than the decision rule, it can be stated that there is some statistical evidence that the threshold has not been reached.

As echinococcosis is commonly a disease of remote, marginalised communities (Craig et al., 2007), surveillance is often hindered by logistical difficulties, and relatively quick and efficient methods are desirable. As such, we used LQAS to evaluate coproELISA prevalence, praziquantel dosing, and local knowledge about echinococcosis in the six communities studied.

### 2.4 Faecal sample collection

A minimum of 19 dogs were sampled in each community (a sample size of 19 minimises the risk of type A and B errors, Valadez et al., 2002), with additional dogs sampled where possible (Bayenoma = 19, Budengjian = 20, Changan Kul = 27, Chahete = 20, Narenhebuke = 21, Tiebukenwusan = 19). Dogs were selected by starting from each community's health centre and walking in a randomly chosen direction (determined by the second hand on a watch) and enquiring about dogs in alternate houses. If dogs were present, these were included in sampling, with ground faecal samples collected from around their owners' houses. If midway through the sampling day it appeared that a minimum of 19 dogs would not be reached by the end of the day, we asked local villagers who served as translators/facilitators to direct us to areas where they knew dogs were present, thus moving away from our chosen random direction. In these areas alternate houses were targeted. The age and sex of each dog was recorded, and dog owners were asked when the dog was most recently dosed with praziquantel. Nine dogs were sampled without their owners present; these dogs were chained and faecal samples were collected from the ground. The sex of these dogs was recorded but no questionnaires were administered. In four communities (Bayenoma, Budengjian, Changan Kul Tiebukenwusan) owners were asked to describe echinococcosis to assess their knowledge about this disease. Questionnaires were administered in Mandarin Chinese, Mongolian or Kazakh depending on the dog owner's native language. Subsamples of faecal samples were stored in 70% ethanol and 0.3% PBS Tween with 10% formalin respectively, and shipped to Salford University at room temperature.

### 2.5 CoproELISA

Faecal samples were extracted by homogenizing, shaking and centrifuging at 2500 r.p.m. (1125 g) for 5 min and collecting the supernatant. Faecal samples were analysed for *Echinococcus* spp. coproantigen with a genus-specific sandwich ELISA using the protocol originally described by Allan et al. (1992) with a modification in that the capture and conjugate antibodies were derived from different rabbit antisera. The conjugate antibody was prepared from hyperimmune rabbit IgG raised against a surface extract from adult *Echinococcus granulosus* worms (Elayoubi and Craig, 2004), and the capture antibody was anti-*E. granulosus* whole worm somatic (Allan et al., 1992). Faecal supernatants of two known positives (an arecoline *Echinococcus* spp. purge positive sample from Kyrgyzstan, and a sample spiked with *E. granulosus* whole worm extract at a 1:100 concentration) were used as positive controls throughout. Two known negative faecal samples from a low endemic area (Falkland Islands) were included as negative controls.

Because Gaussian approaches for calculating ELISA cut-off values (e.g. Allan et al., 1992), are usually based only on a panel of known negatives (often from a non-endemic area) and do not consider the true distribution of both negatives and positives from the population being studied (Gardner and Greiner, 2006), we calculated our cut-off using Receiver Operating Characteristic (ROC) curves (Gardner and Greiner, 2006). All faecal samples collected from necropsied dogs were analysed by coproELISA, and this was treated as a panel of known positives (*n* = 16, with estimated *Echinococcus* spp. worm burdens between 2 and >10,000) and *Echinococcus* spp. negatives, *n* = 22). Using this panel, a coproELISA cut-off of 0.11685 was determined, giving a sensitivity of 94%, a specificity of 77% and an overall accuracy of 84%.

### 2.6 DNA extraction, PCR and sequencing

DNA was extracted from *Taenia* spp. and *Echinococcus* spp. worms using a Qiagen^®^ DNEasy Blood & Tissue kit following the manufacturer's instructions. DNA was extracted from faecal samples using a QIAamp^®^ DNA Stool kit, following the manufacturer's instructions, but using 1 g of faeces. Extracted tissue samples were analysed by PCR using generic cestode primers (von Nickisch-Rosenegk et al., 1999). For the faecal samples it was found that these primers were not suitable, as they cross reacted with non-target DNA (personal observation). Therefore faecal samples were analysed for *Echinococcus multilocularis* (Boufana et al., 2013), and *E. granulosus* (Abbasi et al., 2003; with modifications described by Boufana et al., 2008) using published primers and following described protocols. Positive controls (sequenced DNA from adult *E. multilocularis*/*E. granulosus*/*Taenia. hydatigena*) were used as appropriate for each protocol. Negative controls (PCR grade water) were included in all PCRs. A Stratagene Robocycler (La Jolla, CA) was used for all cycling profiles and PCR products were separated by electrophoresis at 110 V on 1.5% (w/v) agarose gels in Tris–Borate–EDTA buffer (Severn Biotech, UK), stained with GelRed (Cambridge Biosciences, UK). Gels were visualised using Syngene G:Box gel imaging system (Cambridge Biosciences). Tissue samples that were successfully extracted and amplified were sequenced by Beckman Coulter (Essex, UK) and resulting sequences analysed using BLAST (http://blast.ncbi.nlm.nih.gov/Blast.cgi).

### 2.7 Data analysis

The population pyramid for the dog population in Hobukesar and the bar chart of praziquantel dosing were made using ‘package sciplot’ (Morales, 2013) and ‘package pyramid’ (Nakazawa, 2013) in R statistical software, version 2.15.0 (R Development Core Team).

To use LQAS methodology thresholds and corresponding decision rule values must be selected (Valadez et al., 2002). Setting a threshold can be done in several ways, for example a target can be selected (e.g. target for proportion of people vaccinated, etc.), and decision numbers chosen to test whether or not this target has been met. In this case we wanted to assess whether or not the control scheme had led to a reduction in coproELISA prevalence from pre-intervention prevalences. The pre-intervention prevalence was estimated from dog surveys conducted in Hobukesar County prior to the start of the control programme (Wang et al., 2001, 2005). For these surveys, 139 dogs were sampled in Narenhebuke using rectal loops, with the samples tested at Salford University using a similar sandwich coproELISA, for which 50 dogs (36%) were found to be coproELISA positive (Wang et al., 2001, 2005). As the simplified LQAS methodology using tables allowed for setting thresholds to the nearest 5% (Valadez et al., 2002), we conservatively set the upper threshold for coproELISA positive dogs at 35%, to identify communities where the coproantigen prevalence had decreased from this ‘baseline’ value, as would be expected 3 years after the implementation of a dog dosing control programme (WHO, 2011). Decision rule values based on this threshold were estimated (using tables provided in Valadez et al., 2002) as follows: four for Bayenoma (*n* = 19), Budengjian (*n* = 20), Chahete (*n* = 20), Narenhebuke (*n* = 21) and Tiebukenwusan (*n* = 19), and five for Changan Kul (*n* = 27).

To assess whether or not the Echinococcosis Control Programme was reaching households in the local communities, we determined the proportion of dog owners who had dosed their dogs at least once in the 12 months prior to our data collection. We set the threshold at 90%, assuming conservatively that a successful dosing campaign should reach almost all owned dogs at least once a year. Nine dogs sampled without their owners present were excluded from this analysis. The decision rule values were set at 12 for Chahete (*n* = 15), 14 for Bayenoma (*n* = 17), 15 for Narenhebuke and Tiebukenwusan (*n* = 19 each), 16 for Budengjian (*n* = 20), and 21 for Changan Kul (*n* = 27).

In four communities, householders were asked to describe echinococcosis in order to assess people's knowledge of the disease. Studies relating to echinococcosis have been carried out in Hobukesar County previously (Wang et al., 2005) and the National Echinococcosis Control Programme has been carried out in Xinjiang since 2010 (WHO, 2011). We therefore set the knowledge threshold at 65%, i.e. we expected at least 65% of people to be able to describe echinococcosis. As such the decision rule value was set at 7 for Bayenoma (*n* = 13), 8 for Tiebukenwusan (*n* = 15) and 10 for Budengjiang and Changal Kul (both *n* = 19).

## 3 Results

### 3.1 Necropsy panel

Of the 38 dogs necropsied, 20 (52.6%) had *Taenia* spp. and 16 (42.1%) had *Echinococcus* spp. on visual inspection, and 13 dogs (34.2%) were infected with both parasites (Table 1). Only 14 dogs (36.8%) had neither parasite.

A total of 18 *Taenia* spp. tapeworms were collected, but one sample was lost in transport. From the remaining samples, DNA was successfully extracted, analysed by PCR and sequenced, and all 17 were identified as *T. hydatigena* (≥99% match, accession number GQ228819.1). 16 samples of *Echinococcus* spp. were collected, but one sample was lost in transport. For the remaining samples DNA was successfully extracted and amplified, and all 15 were successfully sequenced as *E. granulosus* G1 (≥99% match, accession number DQ408422.1).

### 3.2 Dog demographics and praziquantel dosing

**Fig. 1:**
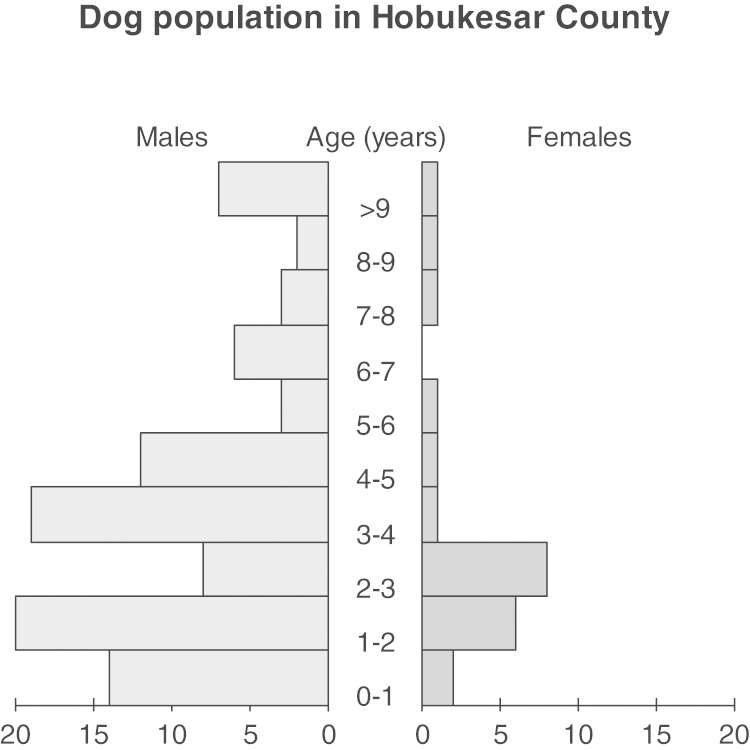
Dog demographics in the six communities sampled in Hobukesar County (*n* = 117 dogs).

A total of 126 owned dogs were sampled in the six communities, with questionnaires administered to 117 owners. The majority of dogs were male (78.6%), and most (72.2%) were 4 years old or younger (Fig. 1).

**Fig. 2:**
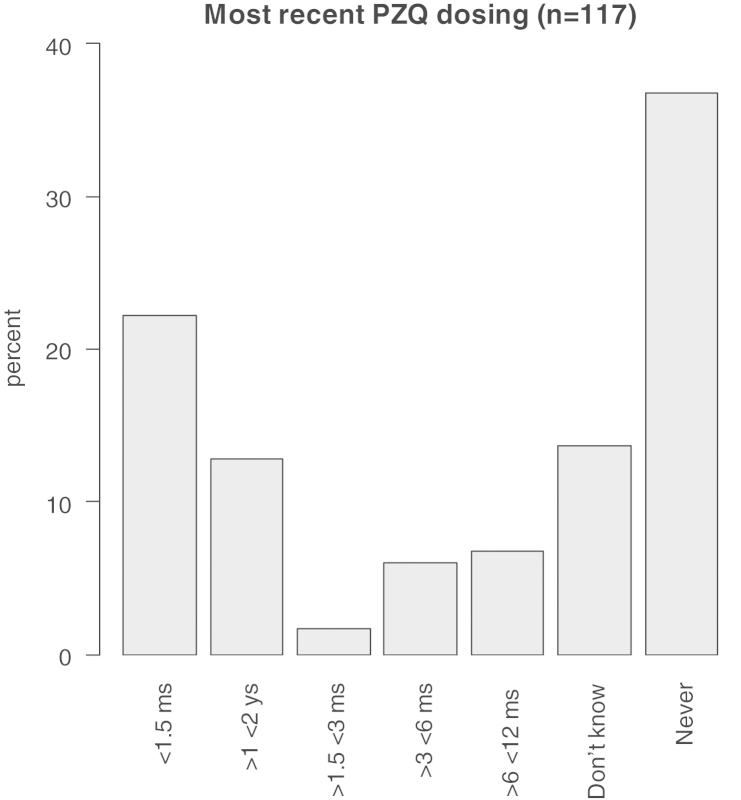
Most recent praziquantel dosing of the dogs sampled in the six communities in Hobukesar County (ms = months, ys = years).

Of the 117 owners questioned, 43 (36.8%) reported never dosing their dogs with praziquantel, and 16 (13.7%) owners did not know when the dog had last been dosed, if ever. Twenty-six dogs (22.2%) were reportedly dosed within the 6 weeks prior to sampling, with others dosed at various times between 6 weeks and 2 years prior to sampling (*n* = 32, 27.4%, Fig. 2; for dosing details per village see Table 2).

In Bayenoma, 13 people were asked to describe echinococcosis and 5 (38.5%) could accurately do so. In Budengjiang 14 of 19 people asked (73.7%) could accurately describe the disease. In Changal Kul and Tiebukenwusan 19 and 15 people were asked about echinococcosis, respectively, with 18 (94.7%) and 4 (26.7%) respondents being able to accurately describe the disease.

### 3.3 Canine echinococcosis in six communities in Hobukesar County

All 126 dog faecal samples were analysed by coproELISA. CoproELISA prevalences ranged from 15.0% in Chahete to 70.0% in Budengjian, with an overall coproELISA prevalence of 41.3% (Table 3).

All coproELISA positive ground faecal samples (*n* = 52) were analysed by coproPCR. In total 26 samples (50%) tested positive for *E. granulosus* DNA. All samples were negative for *E. multilocularis* DNA. Twenty-six samples (50%) were coproELISA positive but coproPCR negative. As these samples were collected from the ground in a relatively dry and warm environment, any DNA in the samples may have been degraded (e.g. Olson et al., 2005), and the presence of PCR inhibitory substances may lead to false negatives (e.g. Mathis and Deplazes, 2006).

### 3.4 Using LQAS to evaluate canine coproELISA prevalence, PZQ dosing and knowledge of echinococcosis in Hobukesar County

The LQAS decision rule for coproELISA positives was met in five of the six communities, with only Chahete having fewer than four coproELISA positive dogs. This provides evidence that the true coproELISA prevalence in Chahete was lower than the 35% threshold. There is no evidence that the true coproantigen prevalence in the other five communities (Bayenoma, Budengjian, Changan Kul Narenhebuke and Tiebukenwusan) was below the 35% threshold.

The decision rule for reported praziquantel dosing scheme coverage over the previous year was only met in Changan Kul where 23 dogs were reportedly dosed in the last year. Therefore, this provides evidence that the praziquantel coverage was lower than 90% in Bayenoma, Budengjian, Chahete, Narenhebuke and Tiebukenwusan.

The decision rule for knowledge of echinococcosis was only reached in Budengjiang and Changal Kul providing some evidence that the level of echinococcosis knowledge was lower than 65% in Bayenoma and Tiebukenwusan.

## 4 Discussion

Cystic echinococcosis is a neglected zoonotic disease that is very difficult to control or eliminate (WHO/OIE, 2001) and to date, only Iceland, New Zealand and Tasmania have declared elimination status for *Echinococcus* spp. (Craig and Larrieu, 2006). Control programmes may include education campaigns, praziquantel dosing of dogs, controlled slaughter (Gemmell et al., 1986), and vaccination of sheep, the intermediate host for *E. granulosus* (Barnes et al., 2012). Echinococcosis Control Programmes are more likely to succeed on islands, where border control is possible and the area targeted is finite and clearly defined (Craig and Larrieu, 2006). However, continental areas present greater challenges for control of echinococcosis, especially regions that are relatively remote and where people are nomadic or semi-nomadic (e.g. Schantz et al., 2003). In these cases frequent praziquantel dosing of domestic dogs (recommended dosing every 6 weeks) may not be practically feasible (Gemmell et al., 1986; Lembo et al., 2013).

In 2006 the Chinese government implemented a National Echinococcosis Control Programme in western China, starting in Sichuan and expanding to other areas including Xinjiang in 2010 (WHO, 2011). It is important to evaluate Echinococcosis Control Programmes and assess how well these are meeting their targets (Craig and Larrieu, 2006; Craig et al., in press). Such assessments are likely to suffer from some of the same challenges as the control programme itself, such as remoteness of communities, logistical challenges and limited time and budgets. Practical assessment tools are therefore highly desirable. We undertook a dog focused assessment of the application and impact of the National Control Programme in Hobukesar County, including dog necropsies, and an LQAS approach to coproELISA tests, and dog owner questionnaires. Whilst the LQAS methodology provides a relatively quick and low-cost assessment tool, it is important to remember that it is not appropriate for estimating prevalences at the village level (i.e. any estimates would be expected to have wide confidence intervals, with the exception of villages where the total number of dogs was comparable to the number of dogs sampled).

We found that of 38 necropsied dogs, 20 (52.6%) had *T.* *hydatigena*, 16 (42.1%) had *E. granulosus*, and 13 (34.2%) dogs had both parasites. Only 14 dogs (36.8%) had neither parasite. Presence of either *Echinococcus* or *Taenia* tapeworms suggests that the dog had not been dosed recently, and had access to livestock offal (Gemmell et al., 1977). The dogs were provided by a local dog catcher, who recorded the location the dogs were sourced, but the exact origin and circumstances of the dogs was not known. Therefore it is important to bear in mind that these dogs are not necessarily representative of the owned dog population, as they were all either stray or unwanted. As praziquantel dosing schemes such as the current one will generally only include owned dogs, stray dogs will not benefit from dosing, and dosing compliance may be lower for unwanted dogs. Furthermore, stray/unwanted dogs may receive less or no food from people, and may be less likely to be restrained and therefore be more likely to scavenge. Stray or unwanted dogs may therefore have higher prevalences of *Echinococcus* and/or *Taenia* spp. infections. Nevertheless, the current findings suggest that active transmission of *E. granulosus* occurs in our study communities, with a high prevalence of canine echinococcosis and taeniasis in the study area.

We used LQAS methodology to investigate three factors related to the success of the control programme: coproELISA prevalence, reported praziquantel dosing, and knowledge of echinococcosis. It is important to note that the coproELISA prevalence is likely to differ from the true prevalence due to limitations in the test sensitivity and specificity. However, as we were only attempting to assess whether the coproELISA prevalence differed from a previous coproELISA estimate, no attempt was made to adjust for this. One challenge associated with LQAS is selecting the thresholds used. In this case, we used data collected from a dog survey in Narenhebuke prior to the start of the control programme (Wang et al., 2001, 2005). This was the only pre-intervention data available from this study area, and we therefore made the assumption that the dogs surveyed in Narenhebuke prior to the control scheme were representative of the dogs in other communities in Hobukesar County. Other surveys in nearby Fuhai and Emin Counties in Xinjiang found that 54/131 dogs surveyed (41.2%) were coproELISA positive (Wei et al., 2005). However, sensitivity analysis found that changing the threshold from 35% to 40% did not affect our results or conclusions (i.e. the same communities would meet or fail to meet the decision number). It should also be considered that the aim of the current study is not necessarily to identify villages which *individually* have experienced a particular reduction in coproantigen prevalence from their own pre-control status, but to identify those villages which currently have a lower coproantigen prevalence than the county-wide ‘average’ pre-control coproantigen prevalence (as individual villages may have had different individual pre-control prevalences). The current approach rather identifies all villages which may be in need of further attention, regardless of the reasons for this. Of the six communities studied, only one (Chahete) showed evidence of having a coproELISA positive prevalence below 35%. Although LQAS identified this village as being different from the other five in meeting the decision number, in this particular case we cannot speak of a reduction in coproELISA prevalence as this community was newly established and would not have existed at the time that Wang et al. ([Wang et al., 2001]2001, [Wang et al., 2005]2005) conducted their surveys. Furthermore, none of the dog owners interviewed in this community reported having dosed their dogs in the previous 2 years (Table 2). This suggests that the relatively low prevalence recorded in this community was unlikely to be due to successful intervention. Chahete was unique in being newly established and based largely on agriculture rather than livestock, which may explain the lower coproELISA prevalence (livestock ownership has been identified as a significant risk factor for human echinococcosis, e.g. Craig and Larrieu, 2006).

In Budengjian and Changan Kul there was no evidence that knowledge of echinococcosis was lower than 65%, and in Changan Kul there was no evidence that the praziquantel dosing rates over the previous year was lower than 90%. However, in both of these villages there was no evidence of a reduction in coproantigen prevalence from the previous estimate (35%). This may be due to infrequent dosing; it is generally suggested that, in order to impact on coproELISA prevalence, praziquantel dosing must be conducted at least four times per year (Lembo et al., 2013).

We found that even modest praziquantel dosing targets (at least 90% of dogs dosed in the previous 12 months) were not met in five communities (Bayenoma, Budengjian, Chahete, Narenhebuke and Tiebukenwusan), and in only one community (Chahete) was there evidence of a reduction in *Echinococcus* spp. coproELISA prevalence to less than the previously recorded 35%. This suggests that the echinococcosis control campaign has had little or no positive impact in these communities.

Although the aims of the Echinococcosis Control Programme, including monthly supervised dosing (Chinese Ministry of Health, 2007), were recommended, it appears that they were over-ambitious in locations such as Hobukesar County, given the associated challenges of the semi-nomadic lifestyles of local people and logistical challenges associated with remote communities. From our data, it appears that sufficiently frequent praziquantel dosing is not being achieved in the communities evaluated. Praziquantel dosing, although highly effective against canine echinococcosis, is often impractical because of the frequent dosing and high proportion of dog coverage required. Although praziquantel rids the dosed dog of worms, it provides no protection against reinfection. Indeed in our samples we found that of the 26 dogs whose owners reported having dosed them no more than 6 weeks prior to sampling, 15 (57.7%) were coproELISA positive. Furthermore, there are other challenges associated with praziquantel dosing, including the fact that dogs dislike the taste and smell of tablets, so that ensuring that the whole dose has been consumed is difficult, as well as difficulties with dosing, as dog weights are usually estimated in the field, and dogs may be under-treated (Larrieu and Zanini, 2012). Therefore, other measures to reduce echinococcosis should be considered.

Dog dosing frequencies of every 6 weeks (eight times a year) are often suggested during a control programme (e.g. Gemmell et al., 1986; Lembo et al., 2013), with the aim of preventing *Echinococcus* spp. from reaching patency even in the case of immediate reinfection (Thompson and McManus 2001), and therefore preventing the release of any eggs from dogs. If this is carried on for a sufficient time period to allow for previously infected intermediate hosts such as sheep to be removed from the population, the transmission cycle of *Echinococcus* can be suspended (see also Larrieu and Zanini, 2012; Torgerson, 2003). However, these dosing frequencies are often not achieved in *Echinococcus* endemic areas (Craig and Larrieu, 2006; Larrieu and Zanini, 2012). As such, it may be better to set more realistic goals; even if it is not feasible to eliminate echinococcosis from a certain area, reductions in transmissions to humans can be achieved with more modest dosing frequencies. For example, mathematical models have suggested that dosing frequencies can be reduced to once every 3 months and still reduce prevalence rates in dogs and livestock to less than 1% within 10–15 years (Torgerson, 2003). It may also be advisable to ensure that supervised dosing of dogs is conducted by trained operatives, rather than relying on dog owners to administer the tablets, as this has been a feature of most successful control campaigns to date, and can help ensure compliance (Craig and Larrieu, 2006).

Previous studies have found that education campaigns could present a practical way of reducing echinococcosis (e.g. Huang et al., 2011). Inclusion of health education has the potential to reduce echinococcosis through increased compliance with dog dosing, a reduction in offal being fed to dogs and/or through improved hygiene, although education alone is unlikely to achieve the desired dosing frequency and decrease in coproELISA prevalence (Craig and Larrieu, 2006; Lembo et al., 2013). Another possible avenue of echinococcosis control is the vaccination of the intermediate host. A safe and effective vaccine against echinococcosis is available for sheep (Heath et al., 2003). Mathematical models suggest that a combination of dog dosing and sheep vaccination is the most effective strategy for echinococcosis control (Torgerson, 2003; Torgerson and Heath, 2003) and vaccination has been successfully trialled in endemic areas (Larrieu et al., 2013). However, there are challenges associated with the vaccine, including the fact that lambs need two doses of the vaccine, and a booster vaccine when they are 1–1.5 years of age (Heath et al., 2003; Larrieu et al., 2013), and the fact that sheep populations are usually much larger than dog populations (Larrieu et al., 2013). This can increase the challenges associated with logistics, although vaccination could be incorporated into other veterinary measures targeting sheep (Heath et al., 2003).

## 5 Conclusions

Our results suggest that the Echinococcosis Control Programme in Hobukesar County in north-west China is still facing several challenges. Although half (50.4%) of all people asked reported dosing their dogs in the last 12 months, the coproELISA prevalence amongst owned dogs remained high in most communities, suggesting little or no reduction has been achieved by the control programme. It is likely that even quarterly praziquantel dosing in these communities is very difficult; they are small rural and remote communities, and many people have semi-nomadic lifestyles that make regular dosing by authorities difficult. The logistical challenges associated with frequent praziquantel dosing and the high coproELISA prevalences found here suggest that additional methods, such as health education and livestock vaccination should be considered to improve compliance levels and the effectiveness of the Echinococcosis Control Programme in Hobukesar County and similar areas. Although many authors agree that elimination of *Echinococcus* spp. from continental areas is often infeasible, attempts to reduce *Echinococcus* spp. transmission should be undertaken in endemic areas where echinococcosis is a public health concern. Instead of aiming to dose dogs every month, which is likely to be overambitious in remote areas, government workers could aim to dose dogs two to four times a year. Public health education could also help reduce transmission to humans, and avenues to integrate sheep vaccination into existing veterinary practices could be explored.

## Funding

This research was funded by the Program for Changjiang Scholars and Innovative Research Team in Universities (IRT1181) and the Wellcome Trust (#094325/Z/10/Z).

## Ethical approval

Ethical approval for this study was provided by the Ethical Review Committee at the First Affiliated Hospital of Xinjiang Medical University, Urumqi, Xinjiang, China.

## Figures and Tables

**Table 1:** Necropsy results (*n* = 38 dogs).

	*Taenia* spp.	*Echinococcus* spp.	*Taenia* spp. and *Echinococcus* spp.
Positive	18 (47.4%)	16 (42.1%)	13 (34.2%)
Negative	20 (52.6%)	22 (57.9%)	25 (65.8%)
Total	38 (100%)	38 (100%)	38 (100%)

**Table 2:** Most recent reported dog dosing with praziquantel in each of the six communities sampled.

Community	No. of dogs sampled	No. of questionnaires administered	No. of dogs reportedly never dosed	No. of dogs with unknown latest dosing	No. of dogs dosed in 6 weeks prior to sampling	No. of dogs dosed >6 weeks to <2 years prior to sampling
Bayenoma	19	18	9 (50.0%)	2 (11.1%)	2 (11.1%)	5 (27.8%)
Budengjian	20	20	1 (5.0%)	5 (25.0%)	6 (30.0%)	8 (40.0%)
Changan Kul	27	27	4 (14.8%)	0 (0%)	12 (44.4%)	11 (40.7%)
Chahete	20	15	14 (93.3%)	1 (6.7%)	0 (0%)	0 (0%)
Narenhebuke	21	19	4 (21.1%)	6 (31.6%)	2 (10.5%)	7 (36.8%)
Tiebukenwusan	19	18	11 (61.1%)	2 (11.1%)	4 (22.2%)	1 (5.6%)
Total	126	117	43 (36.8%)	16 (13.7%)	26 (22.2%)	32 (27.4%)

**Table 3:** CoproELISA positives in each of the six communities sampled. Baye, Bayenoma; Bude, Budengjian; Chan, Changan Kul; Chah, Chahete; Nare, Narenhebuke; Tieb, Tiebukenwusan.

Village	BAYE	BUDE	CHAN	CHAH	NARE	TIEB	Total
Positive	6 (31.6%)	14 (70.0%)	13 (48.2%)	3 (15.0%)	8 (38.1%)	8 (42.1%)	52 (41.3%)
Negative	13 (68.4%)	6 (30.0%)	14 (51.8%)	17 (85.0%)	13 (61.9%)	11 (57.9%)	74 (58.7%)
Total	19 (100%)	20 (100%)	27 (100%)	20 (100%)	21 (100%)	19 (100%)	126 (100%)
